# Ultra-strong and damage tolerant metallic bulk materials: A lesson from nanostructured pearlitic steel wires

**DOI:** 10.1038/srep33228

**Published:** 2016-09-14

**Authors:** A. Hohenwarter, B. Völker, M. W. Kapp, Y. Li, S. Goto, D. Raabe, R. Pippan

**Affiliations:** 1 Department of Materials Physics, Montanuniversität Leoben, Jahnstrasse 12, 8700 Leoben, Austria; 2 Erich Schmid Institute of Materials Science, Austrian Academy of Sciences, Jahnstrasse 12, 8700 Leoben, Austria; 3 Max-Planck Institut für Eisenforschung, Max-Planck-Strasse 1, 40237 Düsseldorf, Germany; 4 Akita University, Tegata Gakuencho, Akita 010-8502, Japan

## Abstract

Structural materials used for safety critical applications require high strength and simultaneously high resistance against crack growth, referred to as damage tolerance. However, the two properties typically exclude each other and research efforts towards ever stronger materials are hampered by drastic loss of fracture resistance. Therefore, future development of novel ultra-strong bulk materials requires a fundamental understanding of the toughness determining mechanisms. As model material we use today’s strongest metallic bulk material, namely, a nanostructured pearlitic steel wire, and measured the fracture toughness on micron-sized specimens in different crack growth directions and found an unexpected strong anisotropy in the fracture resistance. Along the wire axis the material reveals ultra-high strength combined with so far unprecedented damage tolerance. We attribute this excellent property combination to the anisotropy in the fracture toughness inducing a high propensity for micro-crack formation parallel to the wire axis. This effect causes a local crack tip stress relaxation and enables the high fracture toughness without being detrimental to the material’s strength.

The quest for ever stronger engineering materials has a natural upper limit, the so-called theoretical strength, which is around 10–20% of the Young’s modulus^1^,^2^,^3^. Nevertheless, today’s ultimate tensile strength of most engineering materials including iron, nickel, aluminium or titanium-alloys seem to have a much lower, seemingly unsurpassable strength limit around 10% of their theoretical limit^4^,^5^, or 1–2% of the Young’s modulus. The maximum attainable strength and hence the ultimate failure of a material is determined by its resistance against cracks. The damage tolerance of a material, i.e. its capability to sustaining defects without fatal damage, is characterized by its fracture toughness and describes under which loading conditions defects or cracks propagate. To bridge the gap between today’s materials real strength limits and the theoretical strength, better understanding of the fracture toughness is therefore of crucial importance.

However, reviewing material databases a basic conflict between strength and fracture toughness can be observed^6^,^7^. For metallic materials increasing the strength typically leads to the deterioration of the fracture toughness because the materials become more sensitive to defects, which are generally present in metallic engineering materials. This leads to a dilemma where on the one hand, a huge gap remains between the current high strength levels of metallic materials and their actual ultimate strength potential determined by their respective theoretical limits. On the other hand, efforts in increasing the strength by various strengthening mechanisms is associated with a loss in fracture toughness. Consequently, in the material design a compromise between strength and fracture toughness must be widely entered. Today’s strongest metallic bulk material, characterized by its 7 GPa tensile strength, is found in a plain steel first described about 130 years ago^8^. It is a so called pearlitic steel in which two phases, iron and cementite (Fe_3_C), form a lamellar arrangement. Decreasing the inter-lamellae spacing by deformation turns the alloy into a nano-laminate, leading to a drastic increase in strength^9^,^10^,^11^. In the material studied here, which has recently been microstructurally investigated by Li *et al*.^12^, the lamellae spacing is reduced to only a few nanometers and the alloy reaches up to 30% of its theoretical strength. From a microstructural viewpoint the origin of this extraordinarily high strength level can be related to the confinement of dislocation motion^12^,^13^, which is a fundamental strengthening mechanism in nanocrystalline materials^14^,^15^. However, its high damage tolerance is most surprising, since at these high strength levels one would rather expect very low fracture toughness^4^,^5^,^7^. In contrast, without sufficient damage tolerance failure during the synthesis of the material would become very likely. Equally severely, reaching strength levels up to 30% of the theoretical strength could be impeded by crack growth and failure below the microstructural strength limit in the elastic regime.

The main question of this study is how sufficient damage tolerance in terms of fracture toughness can be realized at strength levels far beyond those typical of current engineering materials. To address this question the nanostructured pearlitic steel introduced above was examined by advanced fracture mechanical testing. A clarification could pave the way for the future design of new ultra-strong and damage tolerant engineering materials.

## Material and Experimental Approach

**Figure 1:**
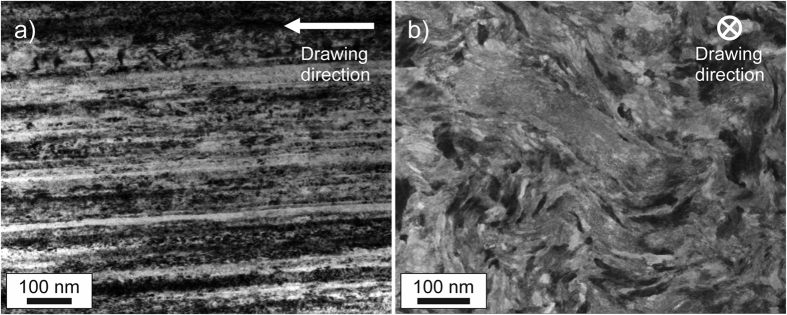
Microstructure of the high deformed state of the wire. (**a**) In the viewing direction perpendicular to the drawing direction the pronounced alignment of the phases and interfaces parallel to the drawing direction is revealed by bright-field TEM. (**b**) View parallel to the drawing direction using STEM exhibiting the characteristic curled microstructure.

Classical pearlitic steels form laminate structures consisting of alternating ferrite and cementite layers. The layers differ in their chemical composition and crystal structure. The ferrite is body-centered cubic iron, whereas the cementite layers consist of Fe_3_C carbides with orthogonal structure in the undeformed state. The wire investigated here was produced by severe cold drawing. The chemical composition is outlined in the Methods section. For comparison two different wires, cold-drawn to true strains of 3.1 and 6.52 with ultimate strengths of nearly 4 and 7 GPa, respectively, were studied. Wire diameters were 120 μm (ε_*log*_ = 3.1) and 24 μm (ε_*log*_ = 6.52), respectively, and we refer to these as the “low” and “high” deformed state in the following. Even though the wire diameters are quite small the samples can be well regarded as a bulk material in comparison with other material types such as powders or coatings owing to its comparably tiny characteristic internal length scales which amount to only a few nm. According to atom probe tomography investigations^12^,^16^ the two wires exhibit differences in the microstructure. In the lower deformed state, with a strength of almost 4 GPa, a nanolamellar structure was found, similar to the one observed in classical pearlitic steels. In the higher deformed states the carbide phase gradually dissolves. The lamellar structure transforms into a nanoscaled carbon-enriched columnar subgrain structure^12^,^17^. TEM-investigations of the wires reveal in both deformation states a strong alignment of the microstructural constituents parallel to the drawing direction, which is exemplarily shown for the 7 GPa wire in Fig. 1a. In the viewing direction parallel to the drawing axis a curled structure can be found, see Fig. 1b. In-depth analyses of the present microstructures can be found elsewhere^12^,^16^.

**Figure 2:**
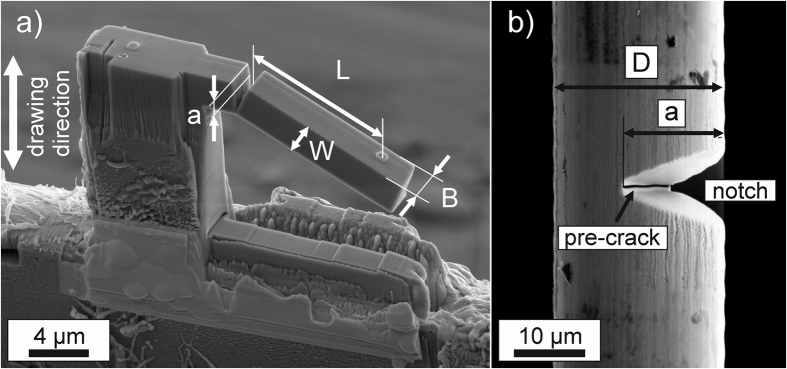
The two different specimen types. (**a**) Micrometer-sized cantilever used for the parallel orientation with indicated sample dimensions, designating the width, *W*, the thickness, *B*, of the beam, crack length, *a*, and the bending length, *L*. (**b**) Single-edge-notched specimen used for the perpendicular orientation with crack length, *a*, and diameter *D*. In both specimen types a fine pre-crack was introduced by focused ion beam milling. The geometry examples originate from measurements on the high deformed state (ε_*log*_ = 6.52), where (**a**) stems from the sample after and (**b**) before the experiment was performed.

To examine the damage tolerance of these wires, orientation sensitive fracture experiments in two different principal testing directions with the crack growth directions parallel and perpendicular to the drawing axis of the wires were defined. For the parallel orientation micrometer-sized cantilevers were fabricated with a focused ion beam (FIB) cutting technique, see Fig. 2a. To produce the micrometer-sized cantilevers large parts of the wire around the actual specimen had to be removed. Therefore, it can be assumed that also first order residual stresses, if any were present in the wire, are eliminated. For the perpendicular orientation single-edge notched tension (SENT) specimens, utilizing in this way the full wire dimensions, Fig. 2b, were used. In both types of specimens the FIB was also utilized to introduce a pre-crack like notch. Since the SENT-specimens did not exhibit bending after the introduction of the pre-crack the extent of residual stresses seem to be rather low.

Due to the small wire dimensions, the experiments with the micrometer-sized cantilevers, probing the fracture resistance parallel to the wire axis, had to be performed inside a scanning electron microscope (SEM). The SEM enables accurate positioning of the specimens with respect to the loading direction and monitoring of crack growth during the experiment. The SENT-specimens could be tested conventionally outside the SEM with a small scale tensile testing machine. For each direction and material state two experiments were performed. From a statistics viewpoint this number is rather small, however, sufficient to confirm the general trends presented in this report. Details on the testing procedures and the consecutive data analysis are outlined in the Methods section. The dimensions of the samples can be found in Supplementary Tables S1 and S2. As will be shown, the fracture properties can be generally described by means of linear elastic fracture mechanics (LEFM), which implies that the presented results are also applicable to larger dimensions of the material that may be synthesized in the future.

## Results and Discussion

### Parallel testing orientation

**Figure 3:**
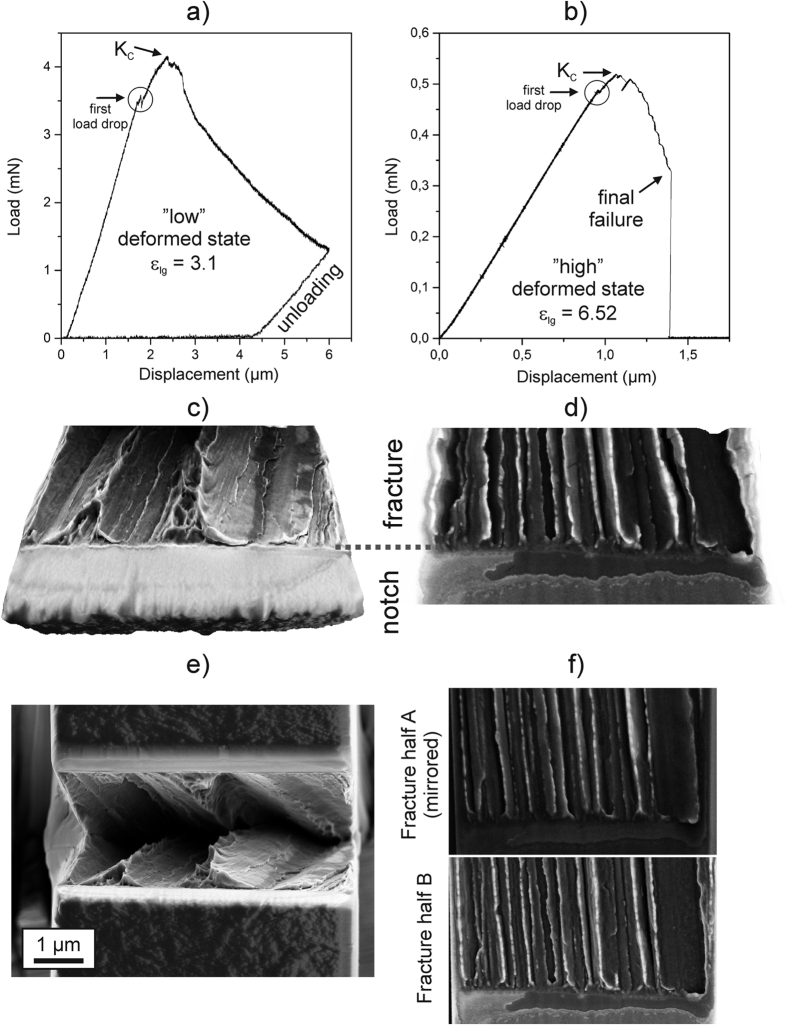
Mechanical test records and typical fractographs presenting significant fracture features in the parallel orientation. (**a**) Load-displacement curve for the low deformed state (ε_*log*_ = 3.1). (**b**) Load-displacement curve for the high deformed state (ε_*log*_ = 6.52). Three-dimensional reconstruction of the fracture surface for the low (**c**) and high deformed (**d**) state. (**e**) Direct comparison of both fracture halves by looking into the broken specimen for the low deformed state. (**f**) Contrasting juxtaposition of both fracture surfaces for the high deformed state. One fracture surface was mirrored horizontally in order to show the complete accordance of both fracture halves. The width of the fracture surfaces, which is equivalent to the full thickness of the specimens is in (**c**,**e**) ~6.2 μm and ~1.5 μm in (**d**,**f**).

Figure 3 shows typical load-displacement curves obtained for the crack growth direction parallel to the drawing axis for both strength levels. At the beginning both types of specimens show pure linear elastic loading, accompanied by a small load-drop, which can be associated with the onset of subcritical crack growth (see videos provided in the Supplementary Material). After reaching the maximum load the force decreases with stable crack growth within the specimens. In the lower deformed state the decrease in load ceases by reaching a maximum pre-defined displacement followed by an unloading sequence, Fig. 3a, whereas the higher deformed state fails catastrophically with a load-drop to zero, Fig. 3b.

To evaluate the fracture toughness for the parallel orientation, the critical fracture toughness, *K*_*IC*_, derived from the maximum load is calculated and summarized in Table 1. The results of a second set of experiments in Table 1 prove the consistency of the results. For this testing direction the fracture toughness is in the range of 5 MPa∙m^1/2^ for the low deformed state and decreases to approximately 4 MPa∙m^1/2^ for the higher deformed material. Calculation of the initiation toughness at the first visible load drop would lead to even somewhat smaller values. On account of the low values of fracture toughness and the high strength levels of the wires the plastic zone size is very small compared to the other dimensions of the cantilevers (see for that also Supplementary) proving that LEFM theory as used here is applicable. Putting the present values in perspective with other materials classes reveals that they are comparable to materials with low fracture toughness such as refractory metals, like pure tungsten^18^, many engineering ceramics^5^ or conventional glasses^5^, which are known as being very sensitive to flaws which renders them unsuited for structural applications. More details to the surprisingly low fracture resistance can be given by fractographic investigations.

Three dimensional reconstructions of the fracture surfaces reveal in both cases, Fig. 3c,d, a distinctive zig-zag fracture surface compared to the flat notch surface. At first sight, this feature could originate from intense local plasticity such as typically observed in ductile materials. However, comparing both corresponding fractured sample portions, Fig. 3e,f, shows that these features mutually match, which excludes strong plastic deformation during crack growth. The fracture morphology therefore represents a rather brittle material behavior which corresponds to the unusually low measured fracture toughness values. The zig-zag structure can be correlated to a curling structure, typically evolving for bcc-iron deformed by wire drawing^19^,^20^. For the low deformed state, Fig 3c, the local smooth surface features combined with the zig-zag structure suggest that the crack follows the lamellar alignment present in the drawn wire. For the higher deformed structure, Fig. 3d, the lamellar structure is principally dissolved according to ATP-measurements^12^. Nevertheless, the fractographs are fairly akin to the low deformed state keeping in mind that the wire diameter between the low and high deformed state is reduced by a factor of 5, which means that the periodicity of the zig-zag structure will decrease in a similar fashion. Even though this testing direction exhibits a very low fracture toughness, ideal brittle fracture would correspond to an even lower fracture resistance. Considering the free surface energy, *γ*_*0*_, with ~2.4 Jm^−2^ referring to experimental data^21^, and the Young’s modulus of 210 GPa for iron, the Griffith toughness^22^ that characterizes the ideal brittle fracture case can be calculated by *K* = (*2γ*_*0*_*E*)^*1/2*^ yielding a value of about 1 MPa∙m^1/2^. Compared to this estimate, the fracture toughness in the present materials is about a factor of 4–5 larger. This discrepancy implies, that at the nanoscale a distinct amount of plasticity during crack growth has occurred even though the fractographs exhibit fairly smooth and well matching features on both sides, Fig. 3e,f. In undeformed pearlitic steels a random orientation of the locally aligned structure (pearlitic colonies) is typically found leading to a tortuous crack path also called cleavage fracture, which has been comprehensively investigated in the last decades^23^,^24^,^25^. This yields for low and moderately deformed materials fracture toughness values higher than those measured here, however, at the expense of distinctively lower achievable strengths^26^,^27^.

### Perpendicular testing orientation

**Figure 4:**
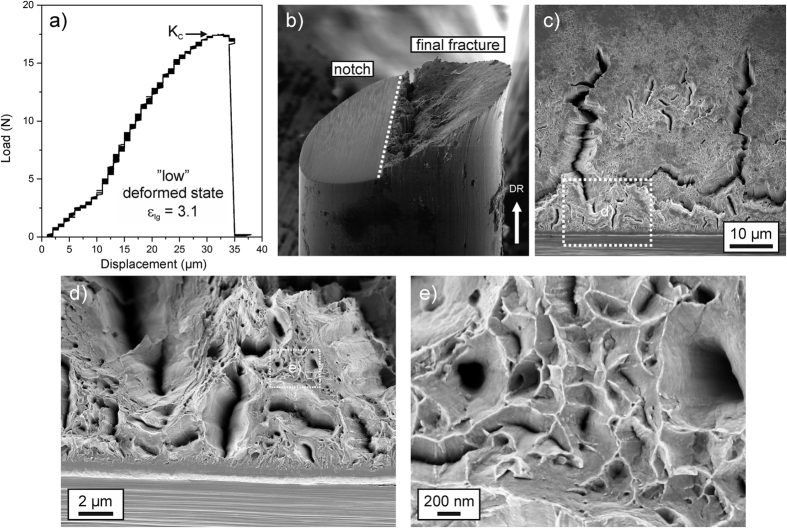
Mechanical test records and typical fractographs presenting significant fracture features in the perpendicular orientation of the low deformed state (*ε*_*log*_ = 3.1). (**a**) Load-displacement curve showing pronounced plasticity before failure. The change in the slope in the elastic regime after about 10 μm is due to an adjustment of the unloaded specimen into the tensile direction. (**b**) Side-view onto a broken sample depicting the pre-notch and the final fracture surface. Sample diameter is 120 μm. (**c**) Typical fractograph looking parallel to the drawing direction. Several large delamination can be observed, which seem to have no specific alignment. (**d**) Enlarged view of inset in (**c**) demonstrating a large variety of smaller delaminations adjacent to the pre-crack. (**e)** Magnified view of detail shown in (**d**). Between the delaminations typical features of ductile fracture with dimples in the range of several hundred of nanometers can be seen.

**Figure 5:**
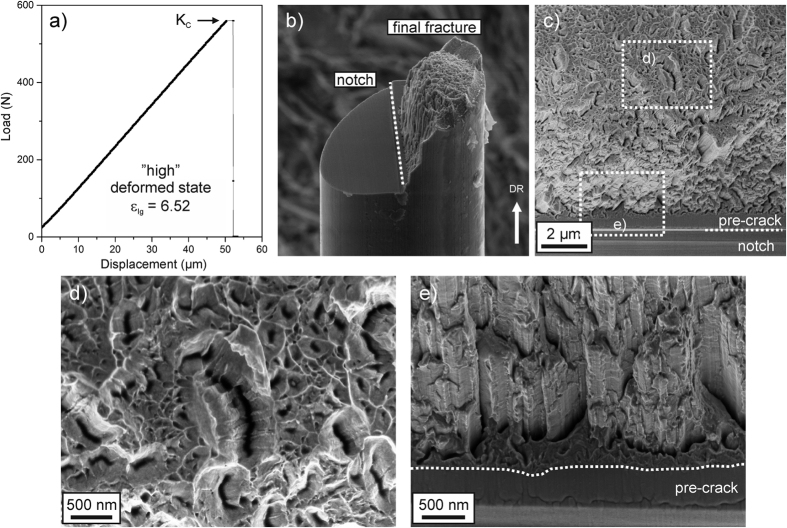
Mechanical test records and typical fractographs presenting significant fracture features in the perpendicular orientation of the high deformed state (*ε*_*log*_ = 6.52). (**a**) Load-displacement curve with pure linear-elastic behavior before failure. (**b**) Side-view onto a broken sample with indicated pre-notch and final fracture surface. Sample diameter is 24 μm. (**c**) Typical fractograph recorded looking parallel to the drawing direction at low magnifications. (**d**) Magnification of detail indicated in (**c**) with ductile fracture features. (**e**) Inclined view onto the transition from pre-crack to final fracture that shows various delaminations before the crack deflects and propagates into the drawing direction. The view is about 30° inclined to the drawing direction to give a better view onto the transition zone with its delaminations and the final crack deflection.

Typical features of the perpendicular testing direction, which is equivalent to the principal loading direction of wires, are compared in Figs 4 and 5. For the low deformed state, before failure a certain non-linearity occurs in the test record, Fig. 4a, which is in contrast to the high deformed state, Fig. 5a, where the response is purely linear elastic. Calculating the fracture toughness from the test records, a value of ~40 MPa∙m^1/2^ is measured for the low deformed state and approximately 20 MPa∙m^1/2^ for the high deformed state. A second set of measurements with comparable results is given in Table 1. To verify and confirm these exceptionally high values the crack tip opening displacement for crack initiation (*CTOD*_*i*_) was measured on the fractured samples of the perpendicular testing orientation. With this SEM-method that is based on three-dimensional reconstructions of the fracture surface, the critical deformation of the crack tip before crack extension takes place can be evaluated and related to the fracture toughness measured from the stress-based K-analysis^28^,^29^. A detailed example of such a measurement and the re-calculation of the corresponding critical fracture toughness in terms of *K*_*IC*_ for both wire types are presented in the Supplementary Material section. The measurements yield values between 38.1 and 43.8 MPa∙m^1/2^ for the low deformed state and results between 17.5 and 20.5 MPa∙m^1/2^ for the high deformed state in the perpendicular testing direction (see Supplementary Table S3). This is in good agreement with the results obtained from the global measurements (Table 1). This result demonstrates the applicability of the linear elastic approach and the agreement with the stress-based analyses suggests that residual stresses do not influence the fracture toughness strongly. In summary, the results show an unusually high difference in the fracture toughness in both materials between the parallel and perpendicular testing orientation, indicating a very strong anisotropy in fracture behavior. To explore the mechanistic origins of the large differences typical fracture surfaces are presented in Figs 4 and 5 for both wire types.

### Fractography

Inspecting the failed sample of the low deformed state, a certain amount of necking can be recognized, Fig. 4b, and secondary cracks have developed, Fig. 4c. These propagate along the wire drawing direction and are referred to as delaminations. Between the large delaminations, smaller ones can be observed at higher magnifications, Fig. 4d. Between the delaminations ductile fracture with dimple rupture is visible, Fig. 4e, which is an indication for good fracture toughness. In the high deformed state, see Fig. 5b, the crack strongly deviates from the anticipated crack path and propagates for a certain length along the drawing direction, i.e. along the direction of the aforementioned low fracture toughness before a smoother final fracture surface is formed. The fracture surface is still micro-ductile, Fig. 5c, and displays again various delaminations, Fig. 5d, surrounded by nano-scaled ductile voids.

As shown in both deformation states the delaminations are a crucial feature and are required for achieving superior fracture toughness: In general, crack fronts carry pronounced tensile stress triaxiality, which controls the deformation of the crack tip and the onset of crack propagation. Calculations show that the maximum stress in this region is about 3 times the yield strength^30^,^31^, see also the Supplementary Materials section. Keeping in mind that the present material in the highly deformed state has a strength of approximately 30% of the theoretical strength, the stresses near the crack tip indeed approach the theoretical limit. However, the theoretical strength is associated with a de-cohesion of the atomic bonds and would lead to brittle cleavage-type fracture with low fracture toughness. Despite this, a relatively high fracture toughness could be measured and can be explained with the occurrence of the delamination zones. Through the occurrence of delaminations the stress component perpendicular to them, and thus the stress triaxiality is reduced leading to higher crack resistance. To be effective for the fracture toughness enhancement delaminations need to form very close to the pre-existing crack, as observed in Figs 4d and 5e. This mechanism leads to globally higher fracture toughness and has also been observed in samples after rolling^7^,^32^ or severe plastic deformation^33^.

A significant aspect for the understanding of these exceptional fracture toughness values is the origin of the delaminations. Figures 4c,d and 5c–e reveal that their crack opening direction is the same as for cracks propagating along the wire axis, which has been found to have an extremely low fracture toughness probed with the parallel orientation measurements. From that it can be rationalized that the weak crack path along the lamella structure with its low fracture resistance is an important requirement for the formation of the delaminations. During loading they are initiated at the weak interfaces and allow a distinctively higher fracture toughness in the principal loading direction as expected from the theoretical viewpoint, suggesting brittle fracture for such ultra-strong materials due to the high stresses at the crack tip close to the theoretical strength.

In addition to the occurring delaminations present in both deformation states, a pronounced crack deflection occurs in the high deformed state, see Fig. 5b. It is a consequence of the aligned microstructure, which exhibits a much stronger crack growth resistance perpendicular to the lamellar structure than parallel to it. This leads to the deflection into the weaker crack growth direction during loading. The real Mode-I fracture toughness would be even higher than the measured K-value as otherwise the crack would not deflect and run into the weak direction. This consideration implies that the results obtained here (around 20 MPa∙m^1/2^) should only be regarded as a lower bound value for the real Mode-I fracture toughness. An upper bound value for this testing direction can be given with the value of the low deformed structure (ε_*log*_ = 3.1), exhibiting a fracture toughness of approximately 40 MPa∙m^1/2^ for this testing direction, see Table 1. This is because with increasing strain the fracture toughness will decrease or stay nearly constant but very unlikely increase. Therefore, the fracture toughness measurements of the low deformed state represent the upper bound value for the real Mode-I fracture toughness in the high deformed state. An inspection of the crack path for the low deformed state, Fig. 4b, demonstrates a slight deflection of the crack as well. Compared to the high deformed state, the deflection only occurs after some global Mode-I crack growth, which determines the fracture toughness. In contrast to the high deformed state, this deflection seems to be an effect of the Mixed-Mode loading conditions introduced by the additional bending torque when the crack becomes very long. To conclude, there is a very fine line between brittle and ductile behavior in such microstructurally nano-scaled highly oriented materials. The accomplishment of high damage tolerance in the main loading direction requires the exploitation of the microstructural anisotropy.

## Conclusions

**Figure 6:**
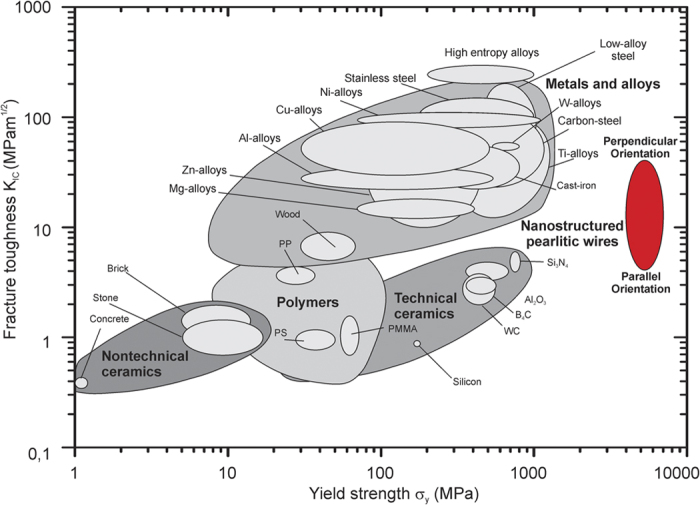
Ashby map presenting the fracture toughness plotted against the yield strength of several important engineering material classes. The investigated steel variants set themselves apart from all other material classes^4^,^5^,^38^ with strength values between 4–7 GPa and fracture toughness values between 4 and 40 MPa.m^1/2^ depending on the testing direction. Even though they do not exceed the fracture toughness of several alloy groups, their damage tolerance in the main loading direction along the wire axis is exceptional and makes them one of the most damage tolerant and strongest materials in the world.

The significance of the results is clearly demonstrated in an Ashby-map, where fracture toughness is plotted against the yield strength, see Fig. 6. The comparison reveals that these nanostructured pearlitic steels exceed in their joint tensile strength plus fracture toughness than any other conventional material class. Even though the fracture toughness of other alloy classes can exceed that of pearlite, the combination of strength and fracture toughness along the loading direction of these pearlitic steel wires is unique. Similar concepts of toughening as presented here are also often discussed in the context of biological and bio-inspired materials^34^,^35^ and often considered as a new design philosophy. With that in mind, it is fascinating to learn that the steel discussed in this study, often regarded as an allegedly “old-fashioned” material, shows such a remarkable damage tolerance and the best known combination of high tensile strength and toughness. The underlying toughening mechanism based on the presence of weak interfaces represents an interesting design concept stimulating further innovative ultra-strong and simultaneous tough metallic alloys.

## Methods

### Material synthesis

The steel has a hypereutectoid composition with 0.98 C, 0.31 Mn, 0.20 Si, 0.20 Cr, 0.01 Cu, 0.006 P, 0.007 S and Fe in balance (wt.%). The steel was austenitized at 1223 K for 80 s followed by the pearlitic transformation, which took place at 853 K for 20 s. This pre-material with an initial diameter of 0.54 mm was then subjected to a cold drawing process. Depending on the diameter reduction, the true (logarithmic) strain amounts to:


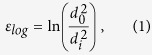


where *d*_*0*_ is the initial diameter and *d*_*i*_ is the reduced diameter of the wire. Two specific states were studied, namely, wires deformed to 3.1 and 6.52 with a thickness of 120 μm (ε_*log*_ = 3.1) and ~24 μm (ε_*log*_ = 6.52), respectively.

### Microstructural investigations

The microstructure was investigated using a JEOL JEM-2200FS TEM (transmission electron microscope) operated at 200 KV in both TEM and scanning TEM (STEM) modes. Samples were prepared using a dual-beam Focused Ion Beam/SEM instrument (FEI Helios NanoLab 600TM) with 30 kV Ga ions and a final low voltage milling at 5 kV Ga ions.

### Sample preparation

Micrometer-sized notched cantilevers, suitable for fracture toughness measurements along the drawing axis, were fabricated using a Zeiss LEO 1540 XB dual beam focused ion beam workstation. From the different wire diameters small sections were lift out for further fabrication steps and adequately positioned onto a FIB lift-out grid, which could be conveniently transferred to the testing stage for the consecutive mechanical experiment. Coarse milling currents of 2 nA to 500 pA were used to fabricate the rough shape, followed by a final polishing of the surfaces with 500–100 pA, in order to reduce the impact of ion damage. Finally, the notch was introduced with a milling current of 50 pA with the ion beam cutting direction parallel to the later loading direction allowing a constant crack depth and notch radius across the thickness of the specimen. From both wire diameters two specimens were fabricated and the characteristic dimensions of the test specimens, including the width, *W*, the thickness, *B*, the crack length, *a*, and the bending length, *L*, are listed in Supplementary Table S1. Due to the large difference in wire diameter the dimension between the two testing conditions along the wire axis are different.

Using the same fabrication process cracks were introduced into the macroscopic specimens used for testing the fracture behavior perpendicular to the drawing axis. For the coarse notch milling currents of 2–5 nA and for the final crack a milling current of 100 pA was in use. The characteristic dimensions, represented by the diameter, *D*, the crack length, *a*, of the crack front are outlined in Supplementary Table S2.

### Sample testing

Due to the different specimen sizes and maximum testing loads various testing machines inside and outside the SEM differing in their maximum load and displacement capacity were utilized to record the mechanical response of the test samples. The fracture experiments accounting for the parallel orientation were performed *in-situ* inside a SEM (Zeiss LEO982). Two different micro indenter system were used. The ASMEC UNAT micro indenter equipped with a diamond indenter tip was the main testing machine for the micro-cantilever experiments. In addition, one experiment was performed with a Hysitron Pico-Indenter (PI-85). The cantilevers were loaded under displacement control to total displacements of 2–6 μm with a constant displacement rate of 1 μm/minute. Videos in terms of SEM-image sequences were recorded, which enables linking the mechanical test record to the crack tip deformation during loading. In total, for each drawing strain and loading condition two experiments were performed, providing consistent results.

The SENT-specimens (single-edge notched tension) of the low-deformed wire sample (ε_*log*_ = 3.1) were tested on a miniaturized testing gear provided from Kammrath and Weiss with a maximum load capacity of 200 N. The SENT-specimens of the high deformed wire (ε_*log*_ = 6.52) were tested on a fibre testing module from the same company with a maximum attainable load of approximately 1 N. The test length of the wires was 1 mm. Similar to the cantilever experiments, described above, for each drawing strain two experiments were performed with consistent results.

### Fracture toughness evaluation

From the load-displacement curves the critical stress intensity, *K*_*Ic*_, was evaluated. The stress intensity of the cantilever specimens used for the evaluation of the fracture toughness parallel to the drawing direction can be calculated according to^36^:


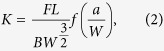


where *F* is the force, taken from test record, *L* the bending length, *B* the thickness of the beam, *W* the width of the specimen and *a* the crack length. From the test record the maximum load, *F*_*max*_, was taken to determine the fracture toughness, K_IC_. The geometry factor *f*(*a/W*) can be written as:


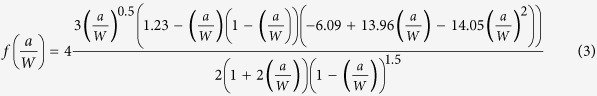


and was derived by using two-dimensional Abaqus finite element simulations^36^.

The stress intensity of the single-edge notched (SENT) specimens subjected to tensile loading conditions used to measure the fracture toughness perpendicular to the drawing axis was evaluated according to:


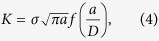


where σ is the applied stress of the round bar with σ = *4F/*(π*D*^*2*^), *F* is the force, *D*, the diameter of the wire and *a* the length of the transverse semi-elliptical surface crack. In each case the maximum load was used to calculate K_IC_. The geometry factor, 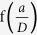, was numerically obtained with the finite element method^37^:


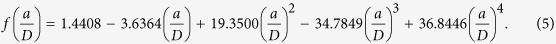


For details concerning the fractographic investigations and video-sequences of the testing process please refer to the Supplementary information.

## Additional Information

**How to cite this article**: Hohenwarter, A. *et al*. Ultra-strong and damage tolerant metallic bulk materials: A lesson from nanostructured pearlitic steel wires. *Sci. Rep.* **6**, 33228; doi: 10.1038/srep33228 (2016).

## Supplementary Material

Supplementary Information

Supplementary Video 1

Supplementary Video 2

## Figures and Tables

**Table 1:** Overview of the collected fracture toughness results for both testing directions.

Specimen	K_Ic, parallel_ (MPa∙m^1/2^)	K_Ic, perpendicular_ (MPa∙m^1/2^)
low deformed-1	5.1	40.1
low deformed-2	4.9	42.5
high deformed-1	3.7	(19.7)
high deformed-2	3.8	(21.1)

Between both specimen orientations the fracture toughness varies between a factor of 5–8. It should be noted that for the high-deformed material with perpendicular specimen orientation the crack strongly deviates from its designated Mode-I crack propagation direction and, hence, cannot be regarded as a pure Mode-I fracture toughness. The calculated values can only be seen as lower bound values for the Mode-I fracture toughness and are therefore put into parentheses.
