# Supplementary material for: Ultra-strong and damage tolerant metallic bulk materials: A lesson from nanostructured pearlitic steel wires
Source: Sci Rep. 2016 Sep 14;6:33228. doi: 10.1038/srep33228 (PMC5021936; doi:10.1038/srep33228)
Supplement: Supplementary Information [file srep33228-s1.pdf]

## Supplementary information for

# Ultra-strong and damage tolerant metallic bulk materials: A lesson from nanostructured pearlitic steel wires

A. Hohenwarter[1,*], B. Völker[1], M. W. Kapp[2], Y. Li[3], S. Goto[3,4], D. Raabe[3], R. Pippan[2]

[1]Department of Materials Physics, Montanuniversität Leoben, Jahnstrasse 12, 8700 Leoben, Austria

[2]Erich Schmid Institute of Materials Science, Austrian Academy of Sciences, Jahnstrasse 12, 8700 Leoben, Austria

[3]Max-Planck Institut für Eisenforschung, Max-Planck-Strasse 1, 40237 Düsseldorf, Germany

[4]Akita University, Tegata Gakuencho, Akita 010-8502, Japan

*** Corresponding author's e-mail address:** anton.hohenwarter@unileoben.ac.at

**a) Fractographic investigations**

The fractography was investigated with a scanning electron microscope (SEM) using a LEO (Zeiss) 1525 equipped with a field emission gun operated with acceleration voltages between 3-20 kV depending on the used detector signal and magnification. As a further measure for the fracture toughness the crack tip opening displacement for crack initiation, $CTOD_i$, was evaluated for the perpendicular orientation in order to compare and confirm the values of the stress based analyses.

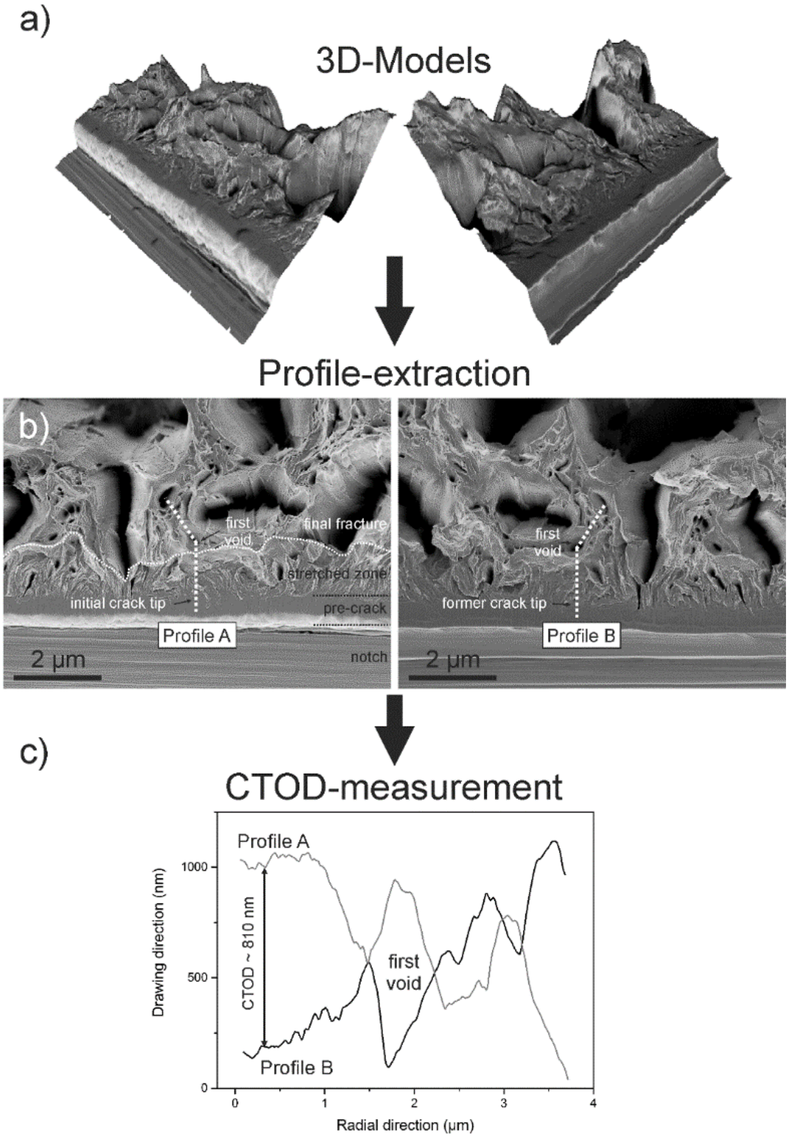


**Figure S1 Detailed *$CTOD_i$*-evaluation.** (a) Three-dimensional surface reconstructions recorded at the same position on both halves of the broken specimen. (b) Comparison of both fracture halves with an indicated arbitrary identical crack path. (c) Extracted profiles from the crack paths and critical arrangement for crack extension at the point of coalescence of the first void with the pre-crack.

This local estimate of the fracture toughness could be directly inferred from the fracture surface, see example taken from the "low deformed" wire in Fig. S1. For Fig. S1, SEM-image pairs were recorded from the same position under different tilt angles on both fracture halves. The area of interest for that was particularily the transition from the pre-crack introduced by FIB-milling to the overload fracture zone. From these images 3-dimensional surface models could be calculated using a software from MeX, Alicona (Fig. S1a). From the 3D-models, representing the exact same area on both fracture halves, arbitrary crack paths taken along identical features on both fracture halves were defined (Fig. S1b). These crack paths deliver height profiles, which allow the reconstruction of the fracture process commencing from the blunting process and to indentify the exact point at which the pre-crack coalesces with the first void or nanocrack ahead of the crack tip, representing $CTOD_i$ (Fig. S1c). Further details regarding the evaluation procedure and the underlying techniques can be found elsewhere [1,2]. The $CTOD_i$, stemming from elasto-plastic fracture mechanic methodologies, can also be used to calculate the equivalent fracture toughness for the linear elastic case according to [3]:

$$K_{IC} = \sqrt{\frac{mCTOD_i \sigma_{UTS} E}{1-v^2}}\,.$$

Depending on the wire-diameter for the ultimate strength 7 or 4 GPa is taken, for the Young's Modulus, *E*, a typical value of 210 GPa, the Poisson's ratio, $\nu$ as 0.3 and for the parameter, *m,* a typical value of 2 for plane strain conditions. The same procedure was also applied for the high-deformed state in the same testing direction and an example is presented in Fig. S2. For both wire-types three single measurements were performed along the crack front and are summarized in Tab. S3. Measurements for the parallel orientation were not performed as theoretical calculations of the $CTOD_i$ would yield values between 5 and 15 nm, which is too small to be measured with this technique.

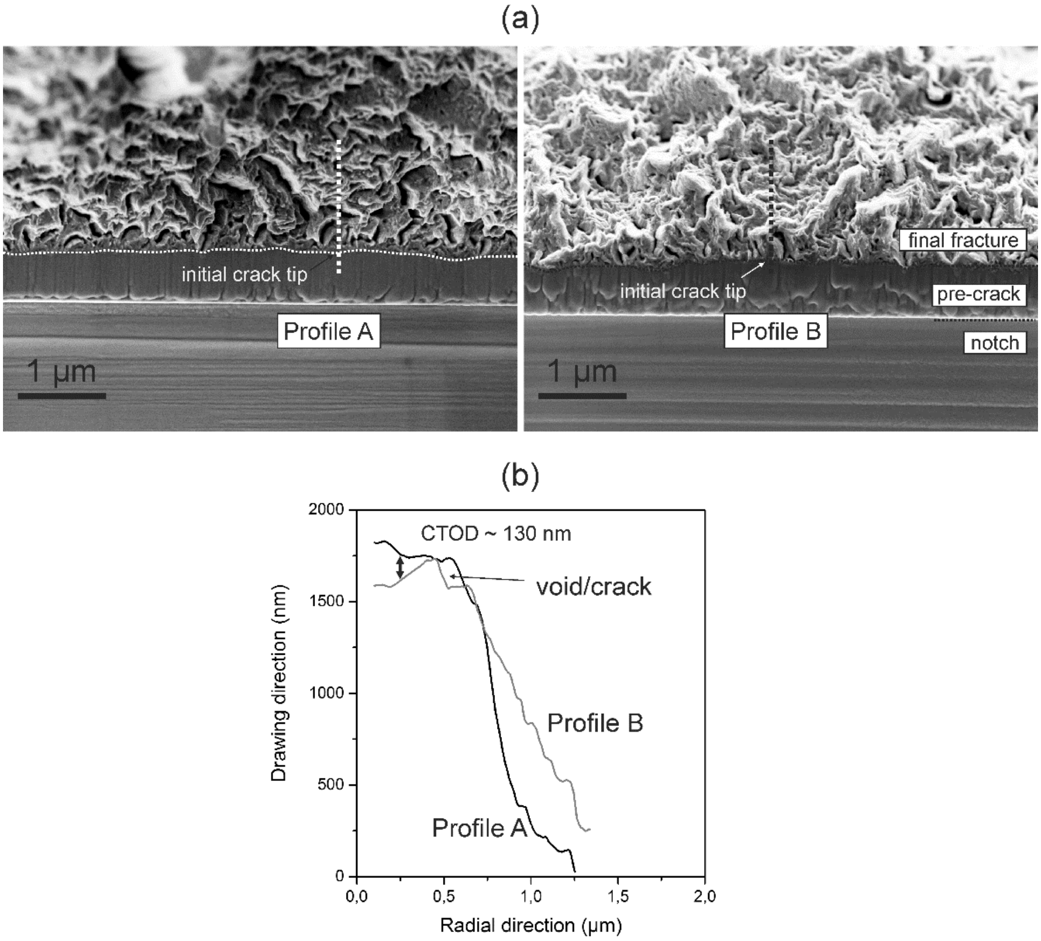


**Figure S2 *CTOD$_i$*-evaluation of the high deformed state.** (a) Comparison of both fracture halves with indicated crack path on both fracture halves. (b) Extracted profiles from the crack paths and critical arrangement for crack extension at which the pre-crack coalesces with the first void or nanocrack ahead of the pre-crack.

**b) Remarks to the stress state ahead of a blunted crack**

For a crack being blunted and assuming a non-hardening material behavior the principal stresses $\sigma_{11}$, $\sigma_{22}$ and $\sigma_{33}$, can be written as[4]:

$$\sigma_{11} = 2\tau_y \ln\left(1 + \frac{2x}{CTOD}\right)$$

$$\sigma_{22} = 2\tau_y + \sigma_{11} = 2\tau_y\left(1 + \ln\left(1 + \frac{2x}{CTOD}\right)\right)$$

$$\sigma_{33} = 0.5\,(\sigma_{11} + \sigma_{22})$$

Here the crack tip opening displacement, *CTOD*, and the yield shear stress, $\tau_y$, has to be taken into account. According to calculations based on flow line theory, the maximum value of $\sigma_{11}$ and $\sigma_{22}$ is reached in a distance of ~ 1.9 *CTOD* ahead of the crack tip[5]. By taking the v. Mises criterion for the equivalent yield strength, $\sigma_y$ with $\tau_y=\sigma_y/\sqrt{3}$, the maximum stresses are:

$$\sigma_{11} \sim 1.8\sigma_y$$

$$\sigma_{22} \sim 3\sigma_y$$

$$\sigma_{33} \sim 2.4\sigma_y$$

The maximum stress $\sigma_{22}$ is much higher than the uniaxial flow stress as a consequence of the multiaxial stress state and is risen by a factor of almost 3 considering a non-hardening material which is a good approximation for the present case. Delaminations reduce $\sigma_{33}$ and as a consequence also $\sigma_{11}$ and $\sigma_{22}$.

**c) Ashby map design**

The Ashby map was partly reproduced from Ashby's book "Material selection in mechanical design" (Butterworth-Heinemann/Elsevier, 2010) page 75. Several major material classes are included and added for High-entropy alloys, where the corresponding data originates from references listed in the main text.

**d) Plastic zone sizes**

For all fracture toughness values the size of the plastic zone was calculated[6] according to Irwin's theory for plane stress conditions as a conservative estimate and included in Tab. S1 and S2:

$$r_{pl} = \frac{1}{\pi}\left(\frac{K_{IC}}{\sigma}\right)^2.$$

In most cases the plastic zone size is much smaller compared to the dimensions of the samples proving the applicability of linear elastic fracture mechanics. The only exception is the perpendicular orientation of the lower deformed wire (D=120 µm), see Tab. S2, where $CTOD_i$-measurements were additionally performed to judge the applicability of LEFM.

**e) Supplementary videos**

2 Supplementary videos are available showing the fracture process of one experiment for the parallel orientation of each wire diameter respectively probing the fracture toughness along the drawing axis. Be aware that in this document only one single frame is visible respectively.

**Video 1 – low deformed wire**

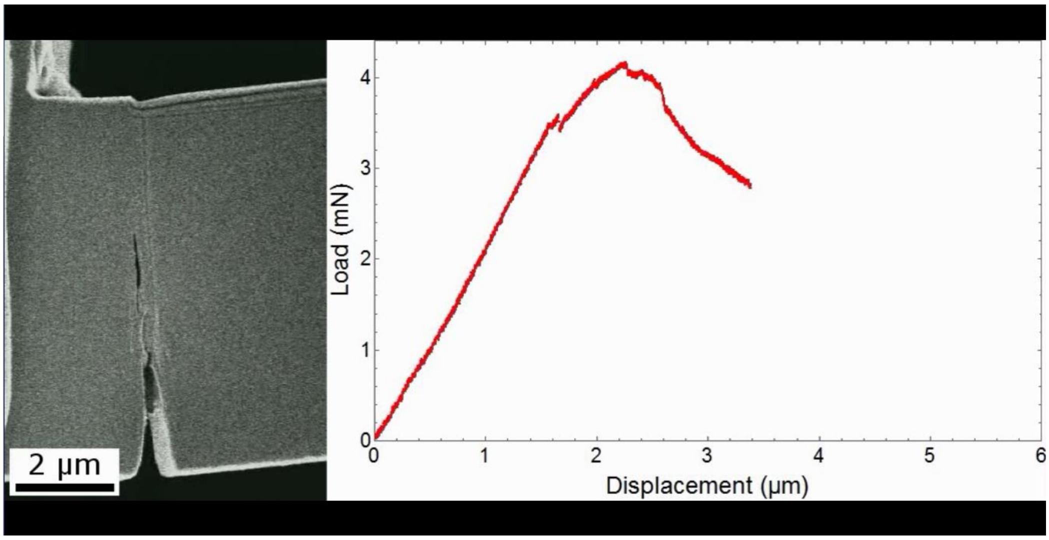


**Video 2 – high deformed wire**

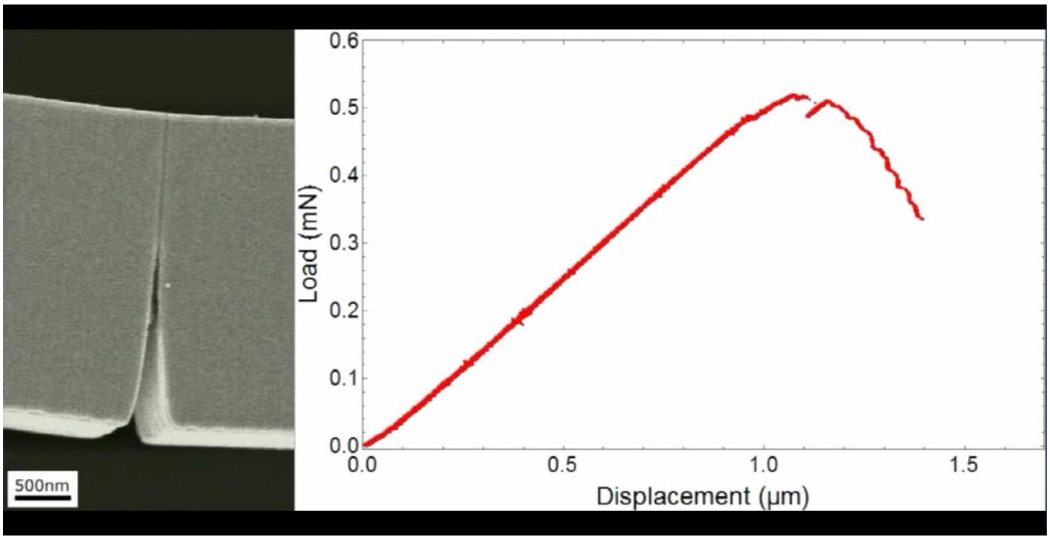

**Supplementary Table S1**

Dimension of the micron-sized notched cantilevers, denoting the wire thickness, *D*, the width, *W*, the crack length, *a*, the thickness, *B*, bending length, *L*, and plastic zone size, $r_{pl}$.

| Sample Nr. | D (µm) | W (µm) | a (µm) | B (µm) | L (µm) | $r_{pl}$ (µm) |
|---|---|---|---|---|---|---|
| 1 | 120 | 7.3 | 1.4 | 6.2 | 28 | 0.510 |
| 2 | 120 | 7.2 | 1.3 | 5.6 | 28 | 0.476 |
| 1 | 24 | 1.8 | 0.7 | 1.5 | 7 | 0.089 |
| 2 | 24 | 2.5 | 0.8 | 2.4 | 10 | 0.092 |

In traditionally larger scaled fracture specimens a geometry ratio, *a/W*, of 0.45 – 0.55 is pursued, but is smaller here for the micron-sized notched cantilevers due to fabrication reasons. Nevertheless, as the plastic zone is small compared to the other dimensions, it should not have an effect on the results.

**Supplementary Table S2**

Dimension of the single-edge notched specimens, denoting the diameter, *D*, the crack length, *a*, and plastic zone size, $r_{pl}$.

| Sample Nr. | D (µm) | a (µm) | $r_{pl}$ (µm) |
|---|---|---|---|
| 1 | 120 | 52.9 | 32.0 |
| 2 | 120 | 54.6 | 35.8 |
| 1 | 24 | 12.4 | 2.5 |
| 2 | 24 | 11.9 | 2.9 |

In the case of the low deformed wires the plastic zone size became comparable to the crack length, however, the additional $CTOD_i$-measurements confirmed the presence of small scale yielding conditions.

**Supplementary Table S3**

Fracture toughness for the crack growth direction perpendicular to the drawing axis in terms of $CTOD_i$ and re-calculated fracture toughness values.

| Wire type | CTOD (µm) | $K_{IC}$ (MPa.m$^{1/2}$) |
|---|---|---|
| low-deformed | 0.81 | 38.7 |
| | 0.79 | 38.1 |
| | 1.04 | 43.8 |
| high-deformed | 0.130 | 20.5 |
| | 0.100 | 18.0 |
| | 0.095 | 17.5 |

**References used in the Supplementary material**


1. Stampfl, J., Scherer, S., Berchthaler, M., Gruber, M. & Kolednik, O. Determination of the fracture toughness by automatic image processing. *Int. J. Fract.* **78,** 35–44 (1996).
2. Stampfl, J., Scherer, S., Gruber, M. & Kolednik, O. Reconstruction of surface topographies by scanning electron microscopy for application in fracture research. *Appl. Phys. A Mater. Sci. Process.* **63,** 341–346 (1996).
3. Anderson, T. L. *Fracture mechanics*. (CRC Press, 1991).
4. Schwalbe, K.-H. *Bruchmechanik metallischer Werkstoffe*. (Hanser Munchen, 1980).
5. Rice, J. R. & Johnson, M. A. *The role of large crack tip geometry changes in plane strain fracture*. (Division of Engineering, Brown University, 1969).
6. Irwin, G. R. Plastic zone near a crack and fracture toughness. in *Proc. Seventh Sagamore Ordnance Mater. Conf. 4* 63–78 (1960).
